# High frequency alternating current neurostimulation decreases nocifensive behavior in a disc herniation model of lumbar radiculopathy

**DOI:** 10.1186/s42234-023-00119-0

**Published:** 2023-07-12

**Authors:** Lauren Savannah Dewberry, Ken Porche, Travis Koenig, Kyle D. Allen, Kevin J. Otto

**Affiliations:** 1 J. Crayton Pruitt Family Department of Biomedical Engineering, University of Florida, 1275 Center Dr. JG56, P.O. Box 116131, Gainesville, FL 32611 USA; 2 Lillian S Wells Department of Neurosurgery at the University of Florida, College of Medicine, 1505 SW Archer Road Gainesville, FL 32608 Gainesville, USA; 3 Pain Research & Intervention Center of Excellence, University of Florida, CTSI 2004 Mowry Road, Gainesville, FL USA; 4 Department of Orthopedics and Sports Medicine, College of Medicine, University of Florida, Gainesville, FL USA; 5 Department of Neuroscience, University of Florida, 1149 Newell Dr. L1-100, P.O. Box 100244, Gainesville, FL USA; 6 Department of Electrical and Computer Engineering, University of Florida, 968 Center Dr, Gainesville, FL 32611 USA; 7 Department of Chemical Engineering, University of Florida, 1030 Center Drive, P.O. Box 116005, Gainesville, FL 32611 USA; 8 Department of Materials Science and Engineering, University of Florida, 549 Gale Lemerand Dr, P.O. Box 116400, Gainesville, FL 32611 USA; 9 Department of Neurology, 1149 Newell Dr, P.O. Box 100236, Gainesville, FL L3-10032610 USA; 10 Nanoscience Institute for Medical and Engineering Technology (NIMET), University of Florida, 1041 Center Drive, Gainesville, FL 32611 USA

**Keywords:** Neurostimulation, High frequency stimulation, Lumbar radiculopathy, Sciatica

## Abstract

**Background:**

The purpose of this study was to evaluate if kilohertz frequency alternating current (KHFAC) stimulation of peripheral nerve could serve as a treatment for lumbar radiculopathy. Prior work shows that KHFAC stimulation can treat sciatica resulting from chronic sciatic nerve constriction. Here, we evaluate if KHFAC stimulation is also beneficial in a more physiologic model of low back pain which mimics nucleus pulposus (NP) impingement of a lumbar dorsal root ganglion (DRG).

**Methods:**

To mimic a lumbar radiculopathy, autologous tail NP was harvested and placed upon the right L5 nerve root and DRG. During the same surgery, a cuff electrode was implanted around the sciatic nerve with wires routed to a headcap for delivery of KHFAC stimulation. Male Lewis rats (3 mo., *n* = 18) were separated into 3 groups: NP injury + KHFAC stimulation (*n* = 7), NP injury + sham cuff (*n* = 6), and sham injury + sham cuff (*n* = 5). Prior to surgery and for 2 weeks following surgery, animal tactile sensitivity, gait, and static weight bearing were evaluated.

**Results:**

KHFAC stimulation of the sciatic nerve decreased behavioral evidence of pain and disability. Without KHFAC stimulation, injured animals had heightened tactile sensitivity compared to baseline (*p* < 0.05), with tactile allodynia reversed during KHFAC stimulation (*p* < 0.01). Midfoot flexion during locomotion was decreased after injury but improved with KHFAC stimulation (*p* < 0.05). Animals also placed more weight on their injured limb when KHFAC stimulation was applied (*p* < 0.05). Electrophysiology measurements at end point showed decreased, but not blocked, compound nerve action potentials with KHFAC stimulation (*p* < 0.05).

**Conclusions:**

KHFAC stimulation decreases hypersensitivity but does not cause additional gait compensations. This supports the idea that KHFAC stimulation applied to a peripheral nerve may be able to treat chronic pain resulting from sciatic nerve root inflammation.

## Background

In 90% of sciatica cases, herniated disc tissue affects the L4/L5 nerve roots (Koes et al. Jun. 2007) by both compressing (Winkelstein et al. Jan. 2002) and causing biochemical inflammation (Kallakuri et al. Dec. 2005; Marshall et al. 1977) around the nerve root and dorsal root ganglion (DRG). During intervertebral disc herniation, a weakened anulus fibrosus allows nucleus pulposus (NP) to extrude from the intervertebral disc. Since the NP is avascular and normally isolated from the immune system, NP extrusion can result in an autoimmune reaction. While NP cells can produce pro-inflammatory cytokines by themselves (Cosamalón-Gan et al. Jan. 2021), causing an inflammatory cascade that results in increased spontaneous neural activity and mechanosensitivity (Takebayashi et al. 1976), glycoproteins found in the NP also act as an antigen that cause antibodies to be produced (Marshall et al. 1977). This activates T lymphocytes (Cosamalón-Gan et al. Jan. 2021) and results in macrophage recruitment to the site (Cosamalón-Gan et al. Jan. 2021). After herniation, activation thresholds are decreased in the impacted nerve (McCarron et al. Oct. 1987), resulting in hyperalgesia and allodynia in the corresponding dermatome (Campbell and Meyer Oct. 2006). This causes a condition known as lumbar radiculopathy, where the affected nerves have lowered activation thresholds.

Clinical management of radiculopathy often includes long-term opioid use, which can lead to opioid use disorder in some patients (Vowles et al. Apr. 2015). Moreover, opioids have limited efficacy for neuropathic pain (McNicol et al. 2013; Furlan et al. May 2006), which is common in lumbar radiculopathy. Other nonsurgical treatments include physical therapy, chemical nerve blocks (Kim et al. Sep. 2016), and epidural steroid injections (Fernandez et al. Nov. 2016). While many patients see symptoms resolve within 8 weeks after a disc herniation, some patients have ongoing neuropathic pain that affects their daily lives (Widerström-Noga et al. Nov. 2001; Hensing et al. 2007; Valat et al. Apr. 2010). These individuals typically undergo surgery to remove herniated tissues. While surgery often provides satisfactory outcomes, re-operation rates are as high as 8% at 2 years and 23% at 10 years. Moreover, follow-up surgeries are less likely to reduce pain and have higher risk of additional complications (Jacobs et al. Apr. 2011). For these reasons, surgery is often delayed in favor of less invasive options. However, additional pain as a result of delaying surgery may contribute to the development of neuropathic pain (Kim et al. Sep. 2016).

Recently, spinal cord stimulation has been used to clinically manage refractory sciatica. Unfortunately, like excision of herniated tissues, surgeries to implant spinal cord stimulators are invasive and carry risks. Moreover, repeated surgeries may be necessary to deal with lead migration, lead fracture, and battery replacement (Osborne et al. Feb. 2011; Kumar et al. Jan. 2007; Deer, et al. 2014; Henderson et al. Jul. 2006; North et al. 2002; Weinstein et al. Feb. 2008; Atlas et al. Apr. 2005; Deyo et al. Nov. 2011).

Peripheral kilohertz frequency alternating current (KHFAC) stimulation can result in a nerve conduction block when applied at a high enough amplitude (Kilgore and Bhadra May 2004; Joseph and Butera Oct. 2011; Joseph and Butera Dec. 2009; Bhadra and Kilgore 2005; Cuellar et al. 2013; Ackermann et al. 2011). At lower amplitudes, peripheral nerve stimulation can facilitate asynchronous firing and result in a decrease in nociceptive behaviors through spinal mechanisms including the gate control theory of pain (Melzack and Wall Nov. 1965; Chung et al. Jul. 1984; Crosby et al. Jan. 2017). KHFAC stimulation could be used for muscle spasticity (Vrabec et al. Jun. 2019), autonomic balance (Pelot and Grill 2020), bladder control (Boger et al. 2008), and chronic pain (Duncan et al. Jul. 2019). For chronic pain, KHFAC stimulation applied to the dorsal root can block or reduce noxious stimuli from the periphery (Cuellar et al. 2013). However, like the spinal cord, the dorsal root can also be difficult to access, and stimulating the dorsal root affects the entire dermatome. Alternatively, KHFAC stimulation of a peripheral nerve could also limit peripheral nociceptive inputs. Even though the inflammation in radiculopathy is at the nerve root, blocking distal to the root could prevent heightened nociceptive signals from reaching the spinal column. For lumbar disc herniation, this could serve as an alternative to opioid use, decrease patient discomfort in movement, and reduce central sensitization during the recovery phase from lumbar disc herniation.

Previously, we showed that KHFAC stimulation distal to a nerve constriction injury can reverse tactile allodynia (Dewberry et al. 2020). While promising, clinical herniation can cause neural sensitization both through mechanical compression and biochemical inflammation. Thus, this study focuses on the inflammatory effect of NP using a model that has less mechanical compression of the nerve root and DRG. Here, NP-induced neuroinflammation is modeled in rats by implanting autologous nucleus pulposus from the tail onto the L5 nerve root and DRG (Shamji, et al. 1976). This approach has the advantage of keeping the L4/L5 spinal column as intact as possible, thereby reducing the loss of disc height at the site of radiculopathy. In this way, the inflammatory actions of the herniated tissue are partially isolated from the mechanical compression of the nerve root. Since KHFAC stimulation has different thresholds for different axon types and voltage gated sodium channel types (Joseph and Butera Dec. 2009; Pelot and Grill 2020; Patel and Butera Jun. 2015; Yi and Grill 2020), it was crucial to examine the ability of KHFAC stimulation to treat inflammatory components of radiculopathy.

## Methods

Radiculopathy was induced via autologous nucleus pulposus implantation on the L5 DRG, with a sham control surgery exposing the DRG. Nerve cuff electrodes were implanted in all animals, with sham electrodes not receiving stimulation. 24 animals had baseline gait and tactile sensitivity measured. 6 of these animals were removed from the study due to post-surgical complications arising from uncured dental cement in the headcap. The remainder were separated into the 3 groups: 1) animals with the NP injury and KHFAC stimulation (NP + KHFAC, *n* = 7, experimental group), 2) animals with NP injury and a sham electrode cuff (NP + Sham, *n* = 6, control), and 3) animals with a sham surgical procedure and a sham electrode cuff (Sham + Sham, *n* = 5, control). Group sizes were selected based on tactile sensitivity effect size from prior experiments (Dewberry et al. 2020). On a surgical day, the groups were selected through random permutation of the group numbers needed using an online random number generator (https://www.random.org/sequences/, which generates randomized sequences of integers). Tactile sensitivity tests were done on days 5, 7, 9, and 13 after surgery. Static weight bearing was measured on days 6 and 16. Gait was recorded on days 7, 11, and 15. On day 7, tactile sensitivity was tested before gait. To accommodate long surgeries and data collection timepoints, animals were split between 2 separate cohorts (*n* = 8 for cohort 1, *n* = 10 for cohort 2), with cohorts separated by 42 days.

Adult male Lewis rats were obtained from Charles River Laboratories (Wilmington, MA) and acclimated to the University of Florida animal care facilities for at least 5 days. Animals were approximately 10 weeks old and weighed approximately 300 g at the beginning of the study. At the time of surgery, animals were 14–20 weeks old Rats were housed in an atmosphere‐controlled room with 1/8-inch corncob bedding, a 12 h light‐dark cycle, and ad libitum access to standard diet and water. Animals had a red shelter and a chew toy for enrichment. Animals were acclimated to behavioral testing enclosures twice for 30 min each. All procedures involving animals were approved by the University of Florida Institutional Animal Care and Use Committee.

### Surgical procedures

Anesthesia was induced using 3% isoflurane and maintained between 1–3% (adjusted based on heart rate). The surgical site was shaved and scrubbed using chlorhexidine and alcohol. Animals were transferred to a heating pad for body temperature maintenance. Saline was injected subcutaneously for hydration (2 ml pre-operation and 3 ml post-operation). For post-surgical analgesia, 0.05 mg/kg buprenorphine was split into pre-operation and post-operation injections, along with 4 additional doses every 8 h.

**Fig. 1:**
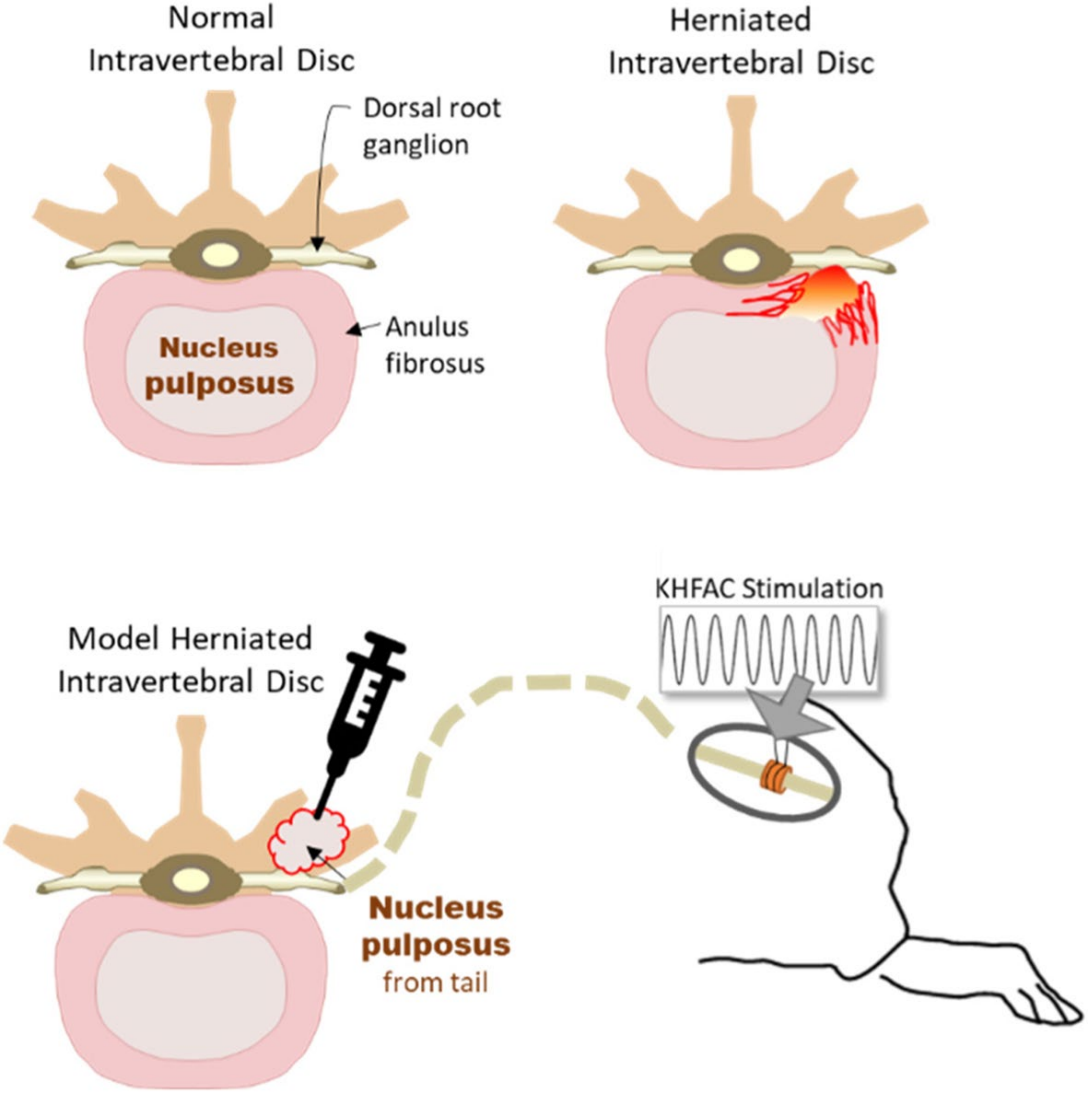
The nucleus pulposus (NP) injury model is achieved by harvesting NP from the tail vertebrae and implanting it on the L5 DRG. Distally on the sciatic nerve, a cuff electrode was implanted to apply HF stimulation

To model herniation, NP was harvested from the tail and placed onto the L5 DRG, as shown in Fig. 1. With the animal prone, a 1 cm incision was made along the length of dorsal surface of the proximal tail. Transversing tendons were retracted to expose a caudal intervertebral disc. The disc was punctured with a 23 G needle and NP was collected and placed into a curette. The site was closed with a single layer closure and 3–0 nylon sutures. The right L5 DRG was then exposed. The line connecting the surface landmarks of the bilateral iliac crests was considered the interspace between the L5 and L6 spinous processes. A 20 mm longitudinal incision was made 5 mm lateral (right) to the midline from the L5 vertebra to the S1 vertebra. The dorsolumbar fascia, which provides attachment to the latissimus dorsi, was opened, and the right paraspinal muscle exposed. The paraspinal muscle was retracted laterally, and then the transverse processes and the lateral surface of the facet joint was exposed. A small rongeur was used to remove the L6 transverse process, which covers the L4 and L5 spinal nerves in a rostral and ventral orientation. Cautery was used as needed. Up to this point the surgeon was blinded to the group. Animals receiving a sham radiculopathy were closed, and NP + animals had the previously collected autologous NP placed onto the DRG before being closed. A two-layer closure was used with 3–0 vicryl sutures for fascia and 3–0 nylon sutures for skin.

To deliver KHFAC stimulation, a cuff was implanted around the sciatic nerve with lead wires tunneled to a headcap connector. To place the cuff, a skin incision on the right leg was made over the biceps femoris of approximately 0.5 cm, parallel to the femur and approximately 1 mm posterior to the external surface of the femur. Muscle bundles were separated to expose the main branch of the sciatic nerve. A 4 mm section of the sciatic nerve was then isolated by blunt dissection, and an electrode cuff was placed on the sciatic nerve. The site was then closed with sutures (4–0 nylon monofilament) and secured with sterile glue if needed. The cuff lead was then secured to the nearby muscle tissue with 4–0 nylon monofilament to prevent pressure on the nerve. After implantation of the cuff electrodes, a rostral-caudal incision (1.5 cm) was made on the skull, and the electrode leads were routed subcutaneously towards the incision. Once the leads were routed, the leg incision was closed. A skull-mounted connector was fixed using four bone screws manually drilled into the skull at points near the lambdoid suture and over the cerebellum. The connector was attached to the cranial screws with acrylic.

Following surgery, animals were recovered from anesthesia on a warm blanket and ability to ambulate was assessed. If an animal was mobile, they were returned to solitary cage housing for 5–7 days, then moved to group housing thereafter. Sutures were removed after 10–14 days.

### HF stimulation procedures

The implanted electrode consisted of a silicone cuff electrode (Microprobes for Life Sciences, Gaithersburg, MD) with 1 mm inner diameter, two platinum-iridium electrode sites spaced 1 mm apart, and with a geometrical surface area of approximately 0.1 mm^2^. The cuff extended 1 mm beyond the electrode contacts at each end. Stimulation was a continuous current driven 50 kHz sinusoid with an amplitude of 1 mA. The stimulation waveform was generated with an optically-isolated stimulus isolation unit (model 2200; A-M Systems) and an electrical circuit to minimize any direct current contamination in the waveform (Franke et al. Jul. 2014).

In our prior work (Dewberry et al. 2020), the blocking waveform was chosen to replicate prior literature; however, this had several limitations. First, direct current offset was observed in the waveform. Direct current can cause a nerve conduction block at low amplitudes (Shannon Apr. 1992), but it can also cause a pH shift near the electrode (Huang et al. Sep. 2001), leading to nerve damage (Shannon Apr. 1992; Agnew et al. Jan. 1989). A capacitor in series with the waveform generator can filter out direct current, but this can lead to charge build up in imperfect capacitors. A better electronic setup is described in Franke et al. (Franke et al. Jul. 2014), with inductors in parallel to shunt charge from the capacitors. We use a similar electrical setup in this work.

Second, an additional limitation of our prior work is that the waveform was voltage-controlled rather than current-controlled. In voltage-controlled waveforms, the charge injected is affected by changes in impedance over time. The impedance of implanted electrodes varies over time due to both biotic factors such as the immune response (McConnell et al. 2009) and abiotic factors like electrode degradation (Sankar et al. 2014). This results in unstable charge injection over time. For this reason, a current-controlled waveform was selected for this work, with an electrical circuit to limit direct current contamination.

### Tactile sensitivity

Tactile sensitivity was evaluated using Chaplan’s up-down method for von Frey fibers (Chaplan et al. Jul. 1994). Animals were placed in von Frey cages to acclimate for 30 min on 3 separate days before beginning the experiment. On testing days, animals were allowed to acclimate for 30 min before connecting the lead to their headcap. Animals were evaluated 3 times per testing day with an approximately 5-min wait between testing periods. All equipment for stimulating was powered on during evaluations, but the headcap lead was connected to the stimulus isolator only during the 2^nd^ evaluation for animals in the experimental group NP + HF Stim.

### Static weight bearing

Static weight bearing was measured to evaluate spontaneous pain and guarding (Schött et al. Apr. 1994). A custom plexiglass incapacitance meter was fashioned to fit existing force plates. The force was calculated based off force plate voltage recorded before, during, and after the rat was placed on the force plates. The position of the animal was monitored via video including use of a mirror placed at 45 degrees below the force plates to ensure each foot was only on one force plate. If the animal was turning or had a foot partially off the force plate, that trial was excluded and reconducted. 3 trials were taken per timepoint, and the accepted trials were averaged. In the NP + HF stim group, 3 trials were taken without and then 3 trials were taken with stimulation.

### Gait

Gait was evaluated using the GAITOR arena (“The Open Source GAITOR Suite for Rodent Gait Analysis | Scientific Reports”. 2019). Briefly, animals were placed in a 6’ × 18″ plexiglass arena with a segmented, instrumented acrylic floor and mirror oriented at 45° underneath the arena floor. On testing days, rodents were allowed to freely explore the arena for 30 min without prompting. When the rat passed through the center of the arena, high-speed video was collected. When a rat’s hind limb strikes an instrumented section of the floor, ground reaction forces are collected.

Videos were analyzed using Deep Lab Cut (version 2.1.8.2) for body part tracking (Mathis, et al. 2018; Nath et al. 2019). We labeled 110 frames taken from 27 videos (95% were used for training). Points were chosen to have minimal effect from skin motion artifact (Lucchetti et al. 1998; Benoit et al. Oct. 2006). Specifically, the nose, feet, and tailbase were labelled along with a point on the apex of the back. The hind feet were labelled at the toe, the 5^th^ metatarsal head, and the ankle. A ResNet-50-based neural network (He et al. 2015; Insafutdinov et al. 2016) was used with default parameters for 500,000 training iterations. The network was validated with 1 shuffle. The test error was 3.89 pixels and the train error was 2.22 pixels (image size was 720 by 480). All the X, Y coordinate pairs were filtered by their ‘likelihood’ value so that only points with likelihoods over 0.9 were included in the analysis. This network was then used to analyze videos from the GAITOR arena. Trials were included only if the animal maintained a steady speed over at least 3 gait cycles (decided a priori).

The Deep Lab Cut results were averaged across strides in a trial. The maximum height of the right (injured) hind heel and toe were analyzed. The horizontal distance between limbs perpendicular to the direction of travel (step width) was measured using the mirror beneath the arena showing a ventral view of the animal. The percent stance time in each stride is reported as a duty factor fraction (Lakes and Allen Nov. 2016). To analyze back hunching, the angle from horizontal to the vector from the tailbase to the back was used. Finally, the flexion of the midfoot was analyzed by finding the angle at midfoot with vectors extending to the hind heel and hind toe.

### Electrophysiologic examination of block

At endpoint, additional microprobes cuff electrodes were placed on either side of the blocking electrode. Additionally, a reference electrode was placed in the tissue nearby. Stimulations were then applied to the distal electrode to elicit compound action potentials. 20–30 stimulations were applied in blocks with and without high frequency stimulation applied. The resulting compound action potentials were recorded with the proximal electrode. Stimulations and recordings were controlled using Synapse software (Tucker Davis Technologies, Alachua, FL).

### Statistical analysis

All statistical analyses were carried out in R (version 4.2.1). Linear mixed model approaches were used for gait analysis, where the baseline measurement and group were treated as fixed effects, timepoint was treated as a fixed effect and repeated measure, and animal identifiers were treated as a repeated measure (Chan et al. Nov. 2022). Velocity was also included as a fixed effect for gait parameters, thereby accounting for velocity effects on raw gait measures. If a parameter varied between limbs (midfoot angle, hind toe height, hind hock height, tailbase to toe angle) then only the limb closest to the camera was evaluated, so limb was used as a factor. For gait, trials were excluded from analysis if the average stride length or average duty factor difference from the predicted value (using the linear model) was greater than 4 standard deviations from the mean. If the linear model indicated an effect of surgery-stimulation, least squared estimated means were compared between specific groups, correcting for multiple comparisons using Tukey’s HSD correction.

For tactile sensitivity, static weight bearing, and end-point electrophysiology measures of activation, a repeated measures ANOVA was used to evaluate between-group differences and pairwise t-tests with Bonferroni correction were used to compare pre-stimulation to stimulated thresholds.

## Results

### Tactile sensitivity decreased during HF stimulation

**Fig. 2:**
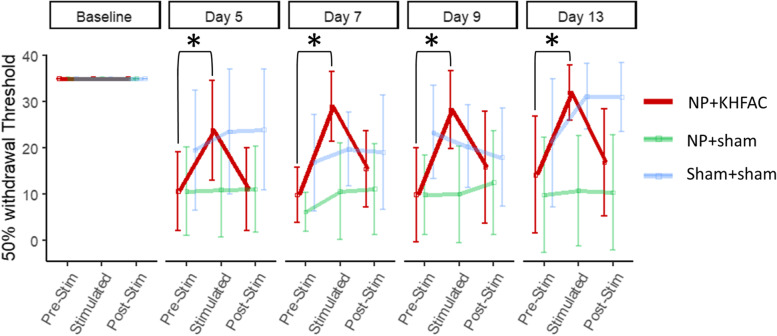
Tactile sensitivity was measured with Chaplan’s up-down method for von Frey filaments. At each timepoint, all animals were tested to find the 50% withdrawal threshold with no stimulation (Pre-Stim), with sham stimulation or KHFAC stimulation (Stimulated), and then again with no stimulation (post-Stim). Shown is the mean ± 95% confidence intervals. Higher values indicate higher withdrawal thresholds and less tactile sensitivity. Both sham stimulation groups showed no significant difference to pre-stimulation (*n* = 6 for NP + Sham, *n* = 5 for Sham + Sham). KHFAC stimulation increased the threshold relative to pre-stimulation for all experimental timepoints (*n* = 7, p < 0.01)

Tactile allodynia is often present in clinical sciatica. Preclinically, it is a measure of evoked pain that correlates to background non-evoked pain (Defrin et al. Dec. 2014). In the present study, all animals showed tactile allodynia after surgery. For all groups, the pre-stimulation 50% withdrawal threshold decreased after injury, indicating increased tactile sensitivity (Fig. 2). Animals with the sham surgery showed less sensitivity relative to animals with NP placement. Increased sensitivity in the sham surgical group is likely due to the invasive sham surgery. The sham surgery involves removal of bone to expose the lumbar DRG and multiple incision sites. Thus, post-surgical tactile sensitivity is not unexpected. However, tactile sensitivity in the sham-sham group was less pronounced than in animals with the NP placement, and sensitivity of the sham-sham group decreased across time while NP injured animals remained sensitized. Additionally, the sham surgical animals recovered over time, as evidenced by increased withdrawal thresholds. The injured animals with sham stimulation never showed effects of sham stimulation and did not have clear drift in thresholds when measured three times in a row. This shows that shifts in tactile sensitivity are not due to a repeated measures effect. No differences were observed with sham stimulation (NP + Sham or Sham + Sham) after normalizing to their respective pre-stimulation threshold.

Animals with KHFAC stimulation had increased withdrawal thresholds only during stimulation (Fig. 2), showing that KHFAC stimulation can treat tactile allodynia. When KHFAC stimulation was applied (NP + KHFAC stimulated), tactile sensitivity increased relative to pre-stimulation levels (*p* < 0.01). In fact, the withdrawal thresholds for stimulated animals were in the range of animals with sham injuries. After stimulation, withdrawal thresholds went back down to the range of sham stimulated animals. Altogether, this data shows that the NP injury caused an increase in tactile sensitivity, and the KHFAC stimulation ameliorated tactile sensitivity associated with NP injury.

### Static weight bearing decreased during HF stimulation

**Fig. 3:**
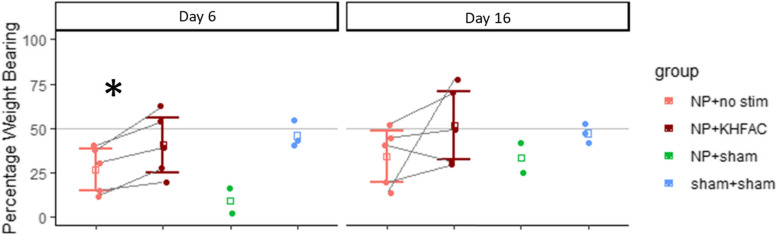
Percent of weight placed on right (injured) hind paw on an incapacitance meter. For NP + KHFAC animals, *n* = 5; NP + sham *n* = 2; Sham + Sham *n* = 3. At six days post-operation, a paired t-test comparing NP + KHFAC animals with and without stimulation showed more weight was placed on the injured limb during stimulation (*p* = 0.0171, *n* = 5). Error bars represent 95% confidence intervals

Weight bearing on the injured limb was decreased with the NP injury but improved during KHFAC stimulation. The weight distribution on left and right (injured) hindpaws were measured while the animals were stationary, as displayed in Fig. 3. Animals without the NP injury placed roughly half their weight on the sham operated limb, but animals with the NP injury and sham stimulation had a lower percentage of their weight placed on the injured limb. This indicates the injury model affected weight distribution and guarding of the injury. The percentage weight bearing was higher on the contralateral (left) foot compared to the injured (right) foot on day 6 for all groups except Sham + Sham (*p* < 0.05). In the experimental group, weight was more evenly distributed between hind limbs when KHFAC stimulation was applied compared to before stimulation. At day 6, weight bearing in animals with NP placement shifted toward a balanced weight distribution during KHFAC stimulation, compared to just prior to stimulation (*p* > 0.05). This trend continued at 16 days after operation, but the shift was not statistically significant at this time point. This indicates that guarding and non-evoked pain are diminished during KHFAC stimulation.

**Fig. 4:**
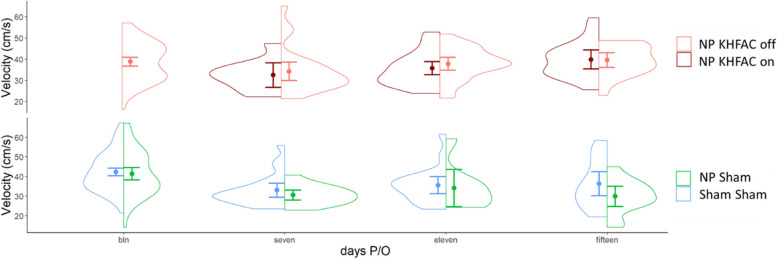
Velocity results over time for all animals are shown. Dots represent mean velocity and lines show 95% confidence intervals. 642 gait trials are included. Since the NP + KHFAC animals are the same in the off and on group, the baseline velocity is only shown once (in the NP + KHFAC off group). KHFAC stimulation did not show a significant effect on velocity

### Gait

Six hundred forty-twotrials were successfully processed for kinematic analysis. Self-selected walking velocity, shown in Fig. 4, differed between groups. In the Sham + Sham group, velocity increased from 33. 40 ± 2.95 [95%CI] at seven days post-surgery to 40.03 ± 6.48 [95%CI] at fifteen days post-surgery. Note, male rats typically increase velocity with repeated exposure to the arena (Steenbergen et al. Jan. 2015; Chan et al. n/d). No significant differences were observed between groups for heel and toe maximum height, step width, duty factor, or back angle (supplement).

**Fig. 5:**
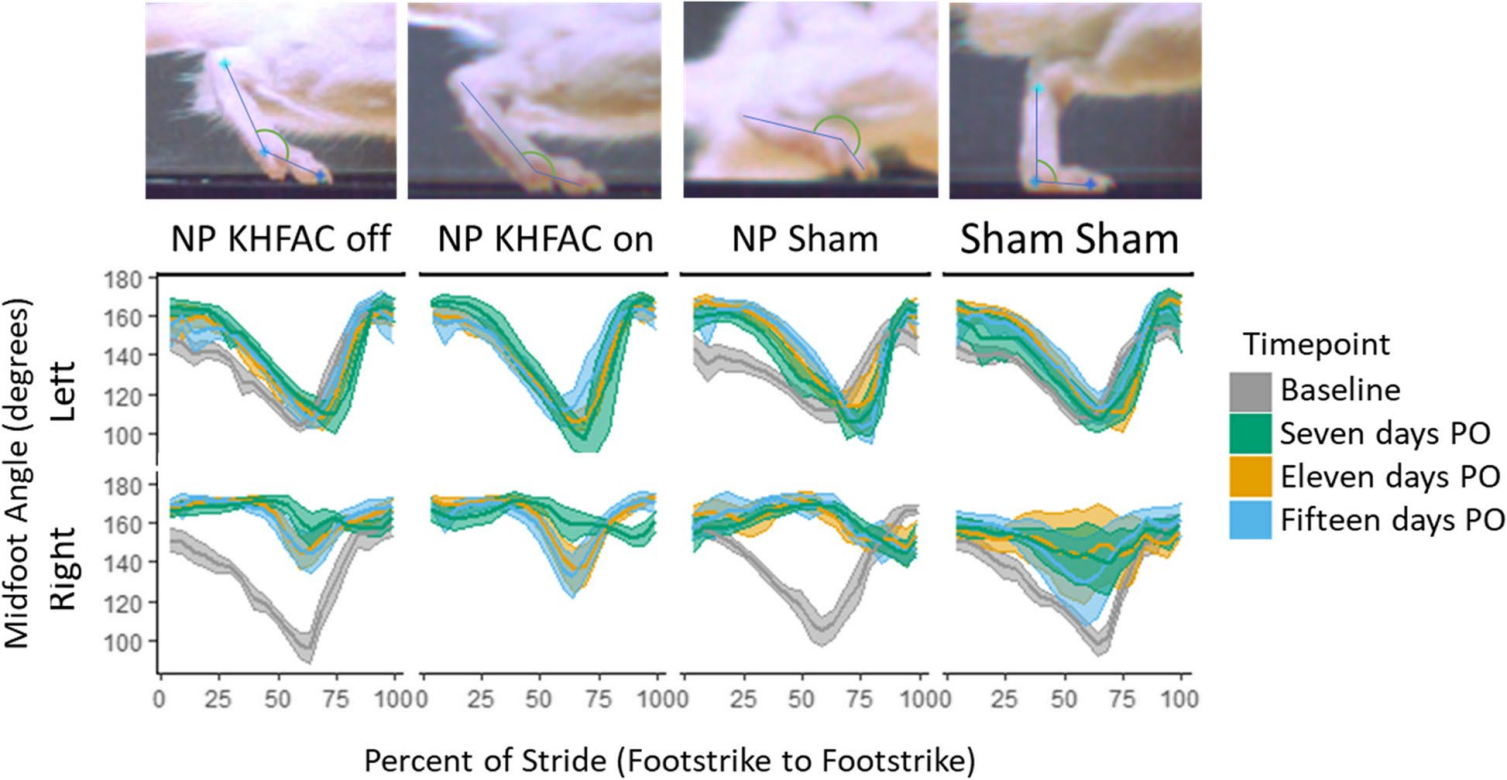
Midfoot angle over stride is shown as the average with bands representing 95% confidence intervals. The average angle between 50 and 75% of stride was compared for each group and timepoint. Midfoot angle differed by surgical group and changed across time (*p* < 0.005) when controlling for velocity. A significant interaction was seen in group and timepoint. Generalized curves were created by averaging all trials for each group on each foot

The flexion of the midfoot was recorded for all conditions, shown in Fig. 5. After NP placement, the midfoot angle between 50–75% of the gait cycle was higher relative to their baseline (*p* < 0.001, indicating less foot flexion near toe-off). There was also some change in the Sham + Sham group relative to its baseline. Again, this is likely due to the invasive sham surgery, and this change was less pronounced than in the other groups that received the NP placement.

Importantly, KHFAC stimulation partially restored midfoot flexion near toe-off, indicated by a visible change of the angle between 50–75% of the gait cycle at 11 and 15 days post-surgery. While this midfoot angle change differed across time, KHFAC stimulation resulted in midfoot angles that were similar to the Sham + Sham control.

### Endpoint electrophysiology

**Fig. 6:**
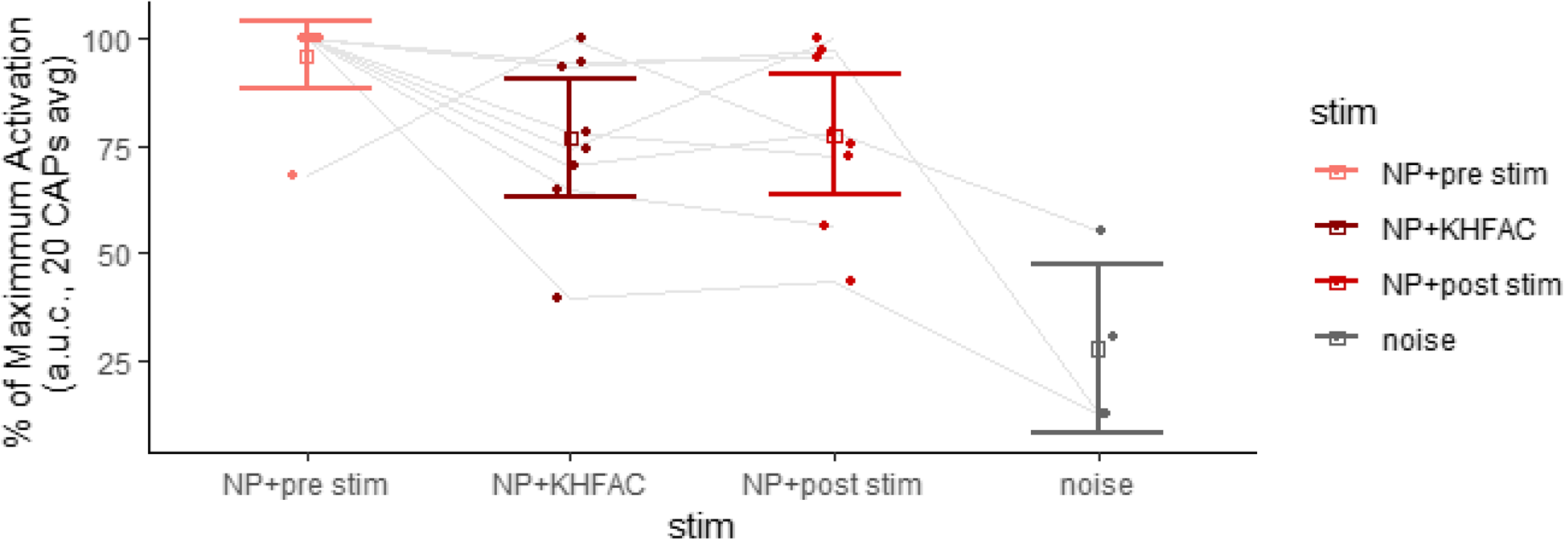
Electrophysiologic measurements of nerve block at end point. For each data point, 20 electrically elicited compound action potentials (CAPs) were recorded and averaged together. For each test block, 20 CAPs before, during, and after KHFAC stimulation were averaged. Animals received 1–2 test blocks. (*N* = 5 animals). Significant differences were observed in an ANOVA on a linear mixed effects model before and during KHFAC stimulation (*p* = 0. 016). Error bars represent 95% confidence intervals

Electrophysiologic measurements of nerve block at end point are shown in Fig. 6. There was a slight decrease between before and during KHFAC stimulation, but this was not significant (*p* = 0.211). Additionally, NP + KHFAC stimulation was higher than noise (*p* = 0.042), indicating a conduction block was not in effect.

## Discussion

Tactile sensitivity, static weight bearing, and midfoot flexion all changed with NP injury, and these same behavioral parameters were improved with KHFAC stimulation. Combined, these data indicate that KHFAC stimulation could be an appropriate treatment option during the early phases of lumbar disc herniation. Importantly, due to multiple surgical sites and removal of bone in the sham surgery, our sham control does show shifts relative to baseline measures. Thus, while KHFAC stimulation may not return behavioral measures to baseline measures, KHFAC stimulation was often able to return behavioral measures to the level of the sham control. This indicates that the KHFAC stimulation may specifically be counter-acting the behavioral effect of NP placement on the nerve root and DRG.

The midfoot flexion results are in agreement with prior literature examining sciatic nerve injuries (Fey et al. May 2010; Varejão et al. Mar. 2004). The midfoot angle change in NP treated animals reflects limited flexion at toe-off. Plantar flexion is controlled by the tibial branch of the sciatic nerve, and dorsiflexion is controlled by the peroneal branch. Decreased dorsiflexion strength is associated with foot drop clinically (Stevens et al. 2015). KHFAC stimulation had marked effects on midfoot angle in NP injured animals. When stimulated, the midfoot angle dropped just prior to toe off, resulting in a shape much more similar to baseline uninjured animals. Interestingly, midfoot flexion was also improved when stimulation was not on. Animals were not stimulated for at least an hour prior to stimulation during gait, so this effect is unlikely to be due to carry over block, which typically stops affecting nerve conduction more quickly after KHFAC cessation (Zhong et al. 2021; Bhadra et al. 2018). Block was applied during tactile sensitivity and static weight bearing tests the day before gait trials, so even the 1^st^ gait timepoint had prior stimulation. Modifications to the central nervous system due to prior KHFAC stimulation sessions may explain the difference in midfoot angle without stimulation.

The stimulation amplitude used in this study was near that used in prior literature (Kilgore and Bhadra May 2004; Patel and Butera Jun. 2015), although some studies have used higher currents (Pelot and Grill 2020). Lower amplitudes of KHFAC stimulation primarily block A neurons on the outside of the nerve (Joseph and Butera Oct. 2011). In mechanical allodynia, A-fiber inputs drive nociception through interneurons in the dorsal horn (Peirs et al. Jan. 2021). Therefore, the improvements seen with KHFAC could be due to blocking of pressure-sensitive Aβ somatosensory neurons that typically carry non-nociceptive signals. Alternatively, some studies indicate that longer application of sub-threshold KHFAC stimulation may be able to block conduction in smaller diameter axons by changing ion concentrations rather than inducing depolarization (Zhong et al. 2021; Zhong et al. May 2022). A robust conduction block was not observed at end point. It is possible the isoflurane anesthesia affected the sodium and potassium channels and interfered with blocking ability (Duch et al. Nov. 1998; Nau 2008). Alternatively, the behavioral effects seen in the present study could be due to stimulation causing increased asynchronous firing, which leads to a decrease in noninfective behaviours through spinal mechanisms including the gate control theory of pain (Melzack and Wall Nov. 1965; Chung et al. Jul. 1984; Crosby et al. Jan. 2017). Finally, synaptic fatigue may play a role (Blitz et al. 2004; Neudorfer et al. May 2021). Synaptic fatigue can cause a lack of response in sensory pathways (Simons-Weidenmaier et al. May 2006) and has a rapid onset at high frequencies (Bowman and McNeal 1986). Clinically, 10 kHz peripheral nerve stimulation showed a significant decrease in neuropathic pain even without complete conduction block (Soin et al. 2015; Finch et al. 2019). Overall, there are multiple potential pathways for the behavioral responses seen. Since a robust nerve block was not observed at end point, it is likely that the stimulation resulted in facilitation of stochastic action potentials in large diameter sensory fibers, leading to inhibition of transmission of small diameter, nociceptive afferents in the spinal cord dorsal horn. Peripheral nerve stimulation results in a decrease in glutamate, substance P, and calcitonin gene related peptide at the spinal cord (Yang et al. Oct. 2022; Sluka et al. Dec. 2005), leading to an analgesic effect. Of note, this model has previously been shown to be TNFα mediated (Allen et al. Aug. 2011). The biological mechanisms of this effect need to be further explored.

There are several considerations of this study that could be improved upon in subsequent studies. A limitation is the all-male study design. Acute studies of KHFAC conduction block in cats do not show differences in blocking thresholds between sexes (Chen 2021; Wang et al. Aug. 2020; “Transcutaneously Coupled, High-Frequency Electrical Stimulation of the Pudendal Nerve Blocks External Urethral Sphincter Contractions - 2009). However, chronic behavioral measurements can be influenced by inflammatory system differences and central processing of stimuli, and are therefore subject to differences between sexes seen in pain processing (Bartley and Fillingim Jul. 2013; Conic et al. Apr. 2022). Additional behavioral assays, like real time place preference (“Behavior”. 2022), could shed more light on whether animals seek treatment over no treatment. Additionally, this injury model does not completely recreate the mechanical compression seen in clinical disc herniation. This was intentional in our study design: Since the effect of KHFAC stimulation on mechanical nerve compression has already been shown (Dewberry et al. 2020), we sought to evaluate KHFAC stimulation as a treatment for the bioinflammatory component of NP in a herniated disc. Finally, KHFAC stimulation of motor neuron conduction could make tactile sensitivity results unreliable due to difficulty in raising or flicking the paw. However, in the present study, a reaction was still seen during KHFAC stimulation, indicating that the increase in 50% withdrawal threshold is likely not an artifact of stimulation. Moreover, the same stimulation was used during gait trials and movement parameters (specifically midfoot flexion angles) improved. Finally, KHFAC stimulation decreased tactile sensitivity while applied, but after stimulation the withdrawal thresholds quickly returned to the range of sham stimulated injured animals. This is typical of high frequency nerve block (Zhong et al. 2021), but not ideal for clinical applications where having a longer treatment effect after stopping stimulation would result in a longer effective battery life.

While the therapeutic aspects of the stimulation are evidenced by the behavioral measures, further investigation into inflammatory changes would clarify the connections between KHFAC stimulation, inflammation, and pain relief. This could be accomplished through robust histological examination with chronic stimulation. In the present study, stimulation was only delivered during behavioral tests. Stimulating for multiple hours every day is likely to result in more pronounced and measurable changes in inflammation. The pathways responsible for these effects likely include pro-inflammatory mediators such as substance P and calcitonin gene related peptide (Yang et al. Oct. 2022). This could be interrogated with inhibitors to negate the KHFAC stimulation effect.

KHFAC stimulation of peripheral nerves may be a useful alternative to spinal cord stimulation when radiculopathy fails to resolve after initial conservative treatment. The durability and sustainability of pain relief with chronic KHFAC stimulation should be investigated. Alternatively, if electrode placement becomes a less invasive procedure (Dalrymple 2021), KHFAC stimulators may be implanted as an early herniated disc treatment to ease pain, decrease opioid dependance, facilitate participation in physical therapy, and decrease the risk of chronic pain development.

## Conclusions

KHFAC stimulation of the sciatic nerve is a promising treatment for lumbar radiculopathy. KHFAC stimulation decreases hypersensitivity and improves gait in animals with an inflammatory nerve root injury. However, a clear nerve conduction block was not seen at end point, so further studies are needed to confirm the mechanism of the treatment. Nonetheless, significant and reproducible behavioral changes were observed, indicating KHFAC is an effective analgesic for inflammatory herniated disc pain.

## Data Availability

The datasets used and/or analyzed during the current study are available from the corresponding author on reasonable request.

## Abbreviations

KHFAC: Kilohertz frequency alternating current
NP: Nucleus pulposus
DRG: Dorsal root ganglion
